# PacBio Long Reads Improve Metagenomic Assemblies, Gene Catalogs, and Genome Binning

**DOI:** 10.3389/fgene.2020.516269

**Published:** 2020-09-08

**Authors:** Haiying Xie, Caiyun Yang, Yamin Sun, Yasuo Igarashi, Tao Jin, Feng Luo

**Affiliations:** ^1^Research Center of Bioenergy and Bioremediation, College of Resources and Environment, Southwest University, Chongqing, China; ^2^PUROTON Gene Medical Institute Co., Ltd., Chongqing, China; ^3^Research Center for Functional Genomics and Biochip, Tianjin Biochip Co., Ltd., Tianjin, China; ^4^The Beijing Genomics Institute (BGI)-Shenzhen, Shenzhen, China

**Keywords:** hybrid assembly, PacBio, gene catalog, anaerobic digestion, genome reconstruction

## Abstract

PacBio long reads sequencing presents several potential advantages for DNA assembly, including being able to provide more complete gene profiling of metagenomic samples. However, lower single-pass accuracy can make gene discovery and assembly for low-abundance organisms difficult. To evaluate the application and performance of PacBio long reads and Illumina HiSeq short reads in metagenomic analyses, we directly compared various assemblies involving PacBio and Illumina sequencing reads based on two anaerobic digestion microbiome samples from a biogas fermenter. Using a PacBio platform, 1.58 million long reads (19.6 Gb) were produced with an average length of 7,604 bp. Using an Illumina HiSeq platform, 151.2 million read pairs (45.4 Gb) were produced. Hybrid assemblies using PacBio long reads and HiSeq contigs produced improvements in assembly statistics, including an increase in the average contig length, contig N50 size, and number of large contigs. Interestingly, depth-based hybrid assemblies generated a higher percentage of complete genes (98.86%) compared to those based on HiSeq contigs only (40.29%), because the PacBio reads were long enough to cover many repeating short elements and capture multiple genes in a single read. Additionally, the incorporation of PacBio long reads led to considerable advantages regarding reducing contig numbers and increasing the completeness of the genome reconstruction, which was poorly assembled and binned when using HiSeq data alone. From this comparison of PacBio long reads with Illumina HiSeq short reads related to complex microbiome samples, we conclude that PacBio long reads can produce longer contigs, more complete genes, and better genome binning, thereby offering more information about metagenomic samples.

## Introduction

Metagenome sequencing represents a powerful approach that allows identification of unknown, non-culturable microbial organisms, discovery of unknown functional genes, and insights into functional processes of specific ecosystems (Parks et al., 2017; Xia et al., 2018; Hua et al., 2019). There are two main sequencing platforms for metagenome sequencing: (i) short-reads sequencing platforms, represented by Illumina, which produce higher-throughput read lengths <500 bp, rarely covering a gene of interest and necessitating assembly before further analysis (Alkan et al., 2010) and (ii) long-reads sequencing platforms, represented by PacBio, which may lead to better contiguity and more complete gene profiling but are limited due to concerns about throughput, cost, and accuracy (Metzker, 2010). Assembly methods that use data from multiple sequencing platforms are rare, and they also risk incorrectly combining sequences obtained from different sequencing platforms.

The error rate of raw PacBio data is 10–15% (Rhoads and Au, 2015), which is difficult to assemble for metagenome, because the error rate may be greater than the genetic difference between organisms, especially for low-abundance organisms. To increase accuracy, the PacBio platform constructs circular consensus sequences (CCS), in which a circular DNA template is read though sufficient sequencing passes before a consensus sequence is reported. A coverage of 15 passes yields >99% accuracy (Eid et al., 2009). Previous research indicates that the use of PacBio CCS provides high-quality long reads that are suitable for metagenomic applications, e.g., hybrid assembly using PacBio CCS and HiSeq contigs produced improvements in assembly statistics, including an increase in the average contig length and number of large contigs (Frank et al., 2016). Alternatively, optimization assembly workflows are adapted for the usage of metagenomic data based on long reads with high error rates. Using a hybrid assembly strategy, combining both long reads from nanopore sequencing and short reads from Illumina sequencing will help to overcome difficulties for assembly and making computation more efficient (Grohmann et al., 2018). Another study demonstrates that it is possible to *de novo* assemble finished metagenome-assembled genomes (MAG) from low-complexity metagenome samples using third generation sequencing data (Somerville et al., 2019). Those studies chiefly focus on assemblies and MAG using single assembly method, but few of those focus on gene catalogs and multiple assembly methods.

As high-throughput sequence-based metagenomics approaches can greatly extend our understanding of microbial functions and their roles in ecosystems, more and more microbial reference gene catalogs for specific ecosystems are being constructed using genomic data. Human gut, ocean, and mouse gut microbial reference gene catalogs have been constructed (Li et al., 2014; Sunagawa et al., 2015; Xiao et al., 2015) and an anaerobic digestion gene catalog with 401,646 genes [open reading frames (ORFs)] has also been constructed (Xia et al., 2018). However, these gene catalogs were based on Illumina short reads and there are no studies on whether PacBio long reads are suitable for constructing gene catalogs.

In this study, we established the feasibility and utility of using PacBio sequencing for metagenomic assembly, gene catalog construction, and genome binning. We evaluated a short-read metagenomic assembler (MEGAHIT), two hybrid metagenomic assemblers (DBG2OLC and OPERA-MS) and depth-based hybrid assembly based on metagenomic data obtained using two sequencing approaches (Illumina HiSeq and PacBio). Integrating PacBio long reads dramatically improved the assembly of large contigs in comparison to only using HiSeq short reads, leading to the assembly of more complete genes and more novel genes. This enhanced the completeness of the genome reconstruction, which was poorly assembled and binned when using HiSeq data alone.

## Materials and Methods

### Samples

Two microbiome samples – a **B**io**f**ilm on Carbon Fiber Filler (BF_M) sample and a Suspended **A**ctivated **S**ludge (AS_M) sample – were obtained from a production-scale biogas fermenter, which operated for 2 months under mesophilic conditions (37°C) with an influent chemical oxygen demand (COD) concentration of 40,000 mg/L. Both samples from the fermenter were rapidly frozen in liquid nitrogen and then stored at −80°C until further use.

### DNA Extraction and Sequencing

Total DNA was extracted from 0.5 g of each sample using a Fast DNA Spin Kit for soil (MP Biomedicals, CA, United States) according to the manufacturer’s instructions with modifications. To obtain a high yield with less DNA degradation, the sludge was homogenized by grinding in liquid nitrogen for 7 min and 0.5 g of the resultant powder was then added to lysis buffer. We constructed Illumina HiSeq DNA libraries for the two samples with an insert size of 250 bp, followed by high-throughput sequencing to obtain 150-bp paired-end reads. High-quality reads were extracted by filtering out low-quality reads (with ambiguous bases or adapter contamination) from the HiSeq raw data. To obtain longer reads, we constructed PacBio DNA libraries for the two samples with an insert size of ∼20 kbp. For quality filtration, reads with a length <50 bp and a quality score <75 were excluded using SMRT Analysis 2.0 software^1^. Sequence data were deposited at the Sequence Read Archive (SRA, NCBI) under Bioproject PRJNA594186.

## Assembly

Various assemblies were performed to compare assembly performance. HiSeq-only reads of each DNA sample were assembled by the MEGAHIT (v1.0.4-beta) assembler (Li et al., 2015) with default parameters. As samples obtained from the same reactor having common microorganism, we merged the two samples for assembly by MEGAHIT (M_MEGAHIT) to construct the HiSeq-only gene catalog (HGC). Hybrid assemblies of MEGAHIT contigs and PacBio long reads were performed using DBG2OLC (Ye et al., 2016) and OPERA-MS (v0.8.0) (Bertrand et al., 2019) with default parameters. Assembly of PacBio long reads only was attempted using Canu (v.1.5) (Koren et al., 2017).

To improve the assembly, we performed three-stage hybrid assemblies of HiSeq and PacBio reads based on the same sequencing depth. The first stage consisted of mapping HiSeq and PacBio reads from AS_M and BF_M to M_MEGAHIT contigs using bowtie2 (Langmead and Salzberg, 2012) and BLASR (Chaisson and Tesler, 2012), respectively, with default parameters. The reads that mapped were divided into 11 phylotypes according to sequencing depth. The HiSeq reads from the same sequencing depth were assembled using MEGAHIT with default parameters. The second stage consisted of pooling the HiSeq contigs with PacBio reads, which underwent hybrid assembly using DBG2OLC (M_depth_DBG2OLC) or OPERA-MS (M_depth_OPERA-MS), with default parameters. Third, after contigs from the samples collected from the fermenter at the same temperature were pooled together and underwent hybrid assembly, redundancy was dealt with using minimus2^2^ and the merged contigs were corrected based on high-quality HiSeq reads.

## Gene Prediction and Construction of the Non-Redundant (NR) Gene Catalog

We used MetaGeneMark (v3.38) (Noguchi et al., 2006) to predict the genes (≥100 bp) in the contigs (≥500 bp) for M_MEGAHIT, M_depth_DBG2OLC, and M_depth_OPERA-MS. Three NR gene catalogs were established by applying the Cluster Database at High Identity with Tolerance (CD-HIT) program (v4.6.5) (Li and Godzik, 2006) to the pooled genes of the three assemblies with a 95% nucleotide identity and 90% length coverage cut-off. The final Reference Gene Catalog (RGC) was established based on the three gene catalogs by applying the CD-HIT program with the same parameters.

Taxonomic assignment of the NR genes was performed by conducting BLASTP searches against the integrated US National Center for Biotechnology Information (NCBI-NR) database with *e*-value ≤1.0×10^−5^. The taxonomic annotation of each gene was then determined by the lowest common ancestor (LCA)-based algorithm using an in-house method (Huson et al., 2007).

### Binning Draft Genomes

Contigs >2.5 kbp in length were grouped into genome bins (GBs) on the basis of abundance and tetranucleotide frequency using MetaBat2 (v2.12.1) (Kang et al., 2019). Completeness and contamination of GBs were assessed using CheckM, which identified lineage-specific single-copy marker genes in each GB (Parks et al., 2015). To reduce contamination of GBs from DBG2OLC contigs, the short and long reads mapped from GB were re-assembled using DBG2OLC. GBs with <60% completeness or >20% contamination were discarded.

### Functional Annotation and Taxonomic Classification of GBs

ORFs of the GB contigs were predicted using Prodigal (Hyatt et al., 2010). The final GBs were annotated using Prokka (v.1.14.5), which is a tool for rapid prokaryotic genome annotation (Seemann, 2014). AMPHORA2 was used to evaluate the taxonomy. A confidence score for each gene was generated at each rank of taxonomic classification (Kerepesi et al., 2014). Marker lineage was reported if 75% of the classifications were in agreement at a particular taxonomic level (Taş et al., 2018).

## Results

### PacBio Long Reads Have Improved Assembly Statistics

We collected two microbiome samples from a production-scale biogas fermenter under mesophilic conditions (37°C). Deep sequencing of these samples was performed on two next-generation sequencing platforms. Using the HiSeq2500 sequencing platform, 150-bp paired-end reads with an insert size of 250 bp were generated, giving approximately 151.2 million read pairs (45.4 Gb). Using the PacBio platform, ∼7.6-kbp PacBio long reads with an insert size of ∼20 kbp were generated, giving approximately 1.58 million long reads (19.6 Gb) (Supplementary Table S1). Regarding the AS_M PacBio read-length distribution, it involved an N50 of 1.2 kbp and >73% of sequences (891,396 reads) were in reads >5 kbp. Regarding BF_M, the values were lower but still sufficient (N50 of 0.8 kbp and >54% of sequences were in reads >5 kbp) (Supplementary Figure S1), making the data suitable for improving assembly contiguity.

**FIGURE 1:**
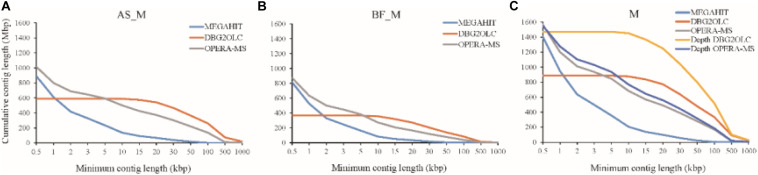
Cumulative number of assembled nucleotides in contigs of different minimum lengths for **(A)** Suspended Activated Sludge (AS_M) sample, **(B)** Biofilm on Carbon Fiber Filler (BF_M) sample, and **(C)** Co-assembly of AS_M and BF_M samples (M). Each line corresponds to a different assembly program (MEGAHIT, DBG2OLC, and OPERA-MS). “Depth” indicates re-assembly according to different sequencing depths.

Various assemblies were performed to compare assembly performance. Overall, we evaluated a short-read metagenomic assembler (MEGAHIT), two hybrid metagenomic assemblers (DBG2OLC and OPERA-MS), and depth-based hybrid assembly based on metagenomic data obtained using two sequencing approaches (Illumina Hiseq and PacBio). Regarding the AS_M sample, MEGAHIT was able to assemble approximately 85.4% of the HiSeq reads generated, which produced 224,876 contigs >1 kb (total contig length: 611 Mbp, 68.6%) with a maximum length of 184,939 bp. The reads of the BF_M sample and the merged samples were also assembled by MEGAHIT, as shown in Supplementary Table S2 and Figure 1. It should be mentioned that very few contigs were >100 kbp when assembled using HiSeq reads only. However, this limitation was addressed by the use of long reads that spanned repetitive sequences in the microbial genomes. The hybrid assembly methods (DBG2OLC and OPERA-MS) using HiSeq and PacBio reads considerably increased the numbers of contigs > 100 kbp, which made up 35 and 10% of the total contig length, respectively, and the assembly contiguity was also improved, particularly regarding the DBG2OLC assembly (N50 increased by approximately 30 times) (Supplementary Table S2 and Figure 1). Notably, DBG2OLC assembly generated 17 contigs >1 Mbp, representing near-complete genomes, with four for OPERA-MS assembly. Comparing the assembly statistics for the two hybrid assemblies showed that DBG2OLC provided more long contigs (>10 kbp) than OPERA-MS, but DBG2OLC assembled a total length of only about half that of OPERA-MS, discarding more reads (Figure 1). Overall, hybrid assembly methods exhibited advantages regarding contiguity and numbers and cumulative nucleotides of contigs >10 kbp., which was also shown by Frank et al. (2016) using HiSeq and PacBio CCS reads. However, hybrid methods have an inherent disadvantage regarding metagenomic assembly.

To improve the assembly, we attempted hybrid assembly using HiSeq short reads and PacBio long reads according to different sequencing depths, as differential coverage binning facilitates the recovery of more complete and higher-fidelity GBs (Albertsen et al., 2013). DBG2OLC depth-based hybrid assembly considerably improved the total contig length, and the HiSeq read mapping ratio increased from 39.45% for DBG2OLC hybrid assembly to 73.24% for DBG2OLC depth-based hybrid assembly (Supplementary Table S2), which indicated that most of the microbial gene information in the biogas fermenter was captured. N50 (57,541 bp) was similar to the N50 (57,140 bp) for the hybrid assembly, and still longer than the N50 reported for recent approaches by other groups including an N50 of 24,610 bp reported by Grohmann’s group (Grohmann et al., 2018) and an N50 of 17,256 bp for the Symbio database (Campanaro et al., 2016; Treu et al., 2016). Interestingly, OPERA-MS depth-based hybrid assembly led to slightly better N50, contiguity, and long contigs than OPERA-MS hybrid assembly. Attempts to perform assembly using only PacBio long reads was ultimately unsuccessful, presumably because of the high error rate (∼15%) and the complexity of the samples. Overall, these results indicate that the incorporation of metagenomic data from the PacBio platform can greatly enhance the genome assembly completeness, especially when DBG2OLC depth-based hybrid assembly is conducted.

### PacBio Long Reads Considerably Improved Gene Catalog Statistics

To evaluate the performance of PacBio long reads regarding constructing a gene catalog, three NR gene catalogs were established based on (1) the merged samples-based MEGAHIT contigs [HiSeq-only Gene Catalog (HGC)], (2) the depth-based OPERA-MS contigs [PacBio OPERA-MS Gene Catalog (POGC)], and (3) the depth-based DBG2OLC contigs [PacBio DBG2OLC Gene Catalog (PDGC)]. Although the total numbers of ORFs was lower for POGC and PDGC than for HGC, the proportion of complete ORFs was improved by 150 and 250%, respectively, compared to HGC (in which 40.29% of the ORFs were complete) (Table 1). Thus, PacBio long-read sequencing led to more complete gene profiling of metagenomic samples, and the ORFs from PDGC were almost all complete (98.86%) (Table 1).

**TABLE 1:** Gene catalog statistics.

**Gene catalog**	**Number of ORFs**	**Total length (bp)**	**Average length (bp)**	**Complete ORFs (%)**
RGC	2,662,347	1,546,177,902	580.76	63.99
HGC	1,889,968	1,094,992,671	579.37	40.29
POGC	1,742,331	1,077,159,972	618.23	60.72
PDGC	1,375,491	681,415,047	495.40	98.86

*HGC, HiSeq-only Gene Catalog; PDGC, PacBio DBG2OLC Gene Catalog; POGC, PacBio OPERA-MS Gene Catalog; RGC, Reference Gene Catalog.*

**FIGURE 2:**
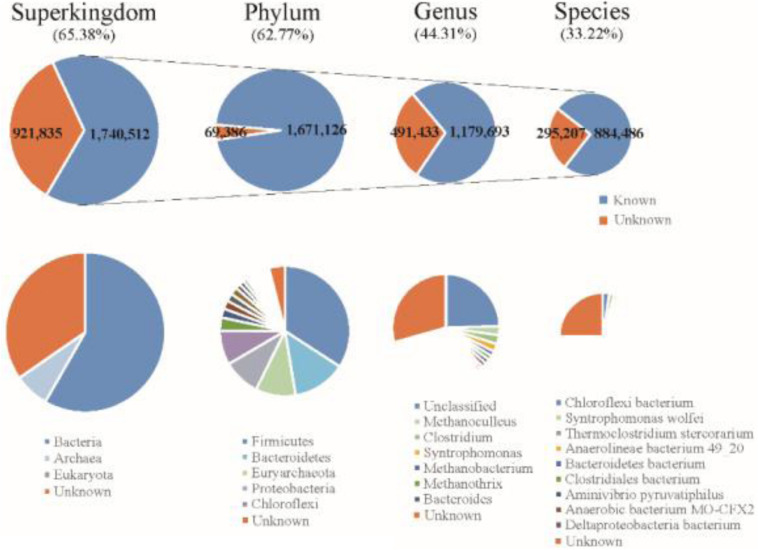
Taxonomic annotation of the RGC to the superkingdom, phylum, genus, and species levels.

The final Reference Gene Catalog (RGC) containing 2,662,347 NR ORFs (63.99% of the ORFs were complete) was then established based on the three gene catalogs. The numbers of ORFs in the RGC had overwhelming advantages compared to published anaerobic digestion gene catalogs, e.g., one gene catalog contained 401,646 ORFs (Xia et al., 2018) and another contained nearly one million (Campanaro et al., 2016). The rate of complete ORFs (63.99%) in the RGC was higher than in the human gut microbial reference gene catalog (57.74%) (Li et al., 2014). Based on known sequences from the integrated US National Center for Biotechnology Information (NCBI-NR) database, 1,551,890 ORFs (58.29%) of gene catalog RGC could be classified to bacteria and 187,348 ORFs (7.04%) could be classified to archaea using the lowest common ancestor approach (Figure 2). According to the taxonomical distribution in gene catalog RGC, Firmicutes (22.32%) dominated the known taxa at phylum level, followed by Bacteroidetes (8.66%), Euryarchaeota (6.52%), Proteobacteria (6.04%) and Chloroflexi (5.52%); *Methanoculleus* (1.72%), *Clostridium* (1.69%), and *Syntrophomonas* (1.49%) dominated at genus level. However, 409,872 ORFs (15.40%) could be classified to metagenome-assembled genomes or unclassified species and did not know come from which genus.

**FIGURE 3:**
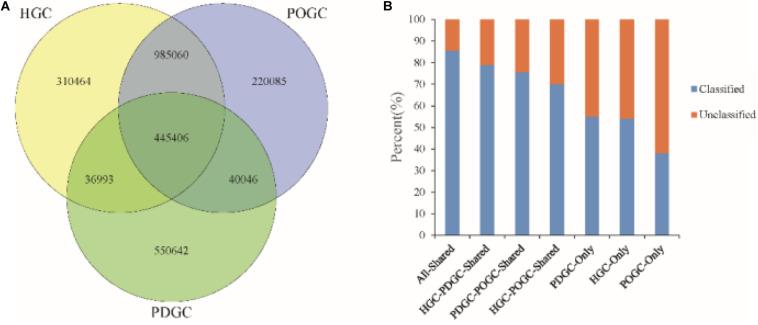
Characteristics of gene catalogs constructed from different assemblies. **(A)** Venn diagram of genes of the final Reference Gene Catalog (RGC), which was established based on the MEGAHIT short reads gene catalog [HiSeq-only Gene Catalog (HGC)], the DBG2OLC depth-based hybrid assembly gene catalog [PacBio DBG2OLC Gene Catalog (PDGC)], and the OPERA-MS depth-based hybrid assembly gene catalog [PacBio OPERA-MS Gene Catalog (POGC)]. **(B)** Percentage of classified genes in the HGC, PDGC, POGC, and combinations.

We also analyzed different assembly methods for constructing gene catalogs. 445,406 ORFs (16.73% of the RGC) were shared among the three gene catalogs, of which 85.54% were classified, which was a higher percentage than in the RGC (65.38%). 83.57% of ORFs in the HGC were redundant, of which 82.27% (1,299,440) were removed due to incompleteness. The advantage of hybrid assembly was also seen by the fact that more genes were assembled, with POGC and PDGC providing an additional 220,085 and 550,642 ORFs (Figure 3A), respectively, thereby offering more functional information about the metagenomic samples. Most of the ORFs could not be classified (∼50%) (Figure 3B), indicating that many functional genes and organisms in biogas fermenters are still unknown, and the approaches used in this study could greatly extend our knowledge in this field.

To assess the quality of the catalog, we mapped short-reads from Illumina HiSeq platform to the gene catalog RGC. An average of 77.92% of HiSeq sequencing reads could map to the RGC, indicating that we captured most of the microbial gene information in the biogas fermenter. Interestingly, 309,605 ORFs were mapped none of the short-reads, of which 228,478 ORFs (73.8%) come from PDGC-Only, further highlighting advantages over short-read and adding long-read metagenome assembly for finding unknown genes. The results further indicate that hybrid metagenomic assembly has advantages over short-read-only metagenomic assembly regarding genetic integrity and producing more genes.

### PacBio Assemblies Produce More Complete Genomes

Genomes are essential for improving our understanding of microbial evolution and ecology (Albertsen et al., 2013). Culture-independent molecular approaches can provide information on taxonomic classification and the functional roles of microbes in ecosystems, bypassing the cultivation bottleneck (Albertsen et al., 2013; Stolze et al., 2016), e.g., by binning of metagenome contigs of unknown and distinctive taxa. MetaBat2 was used to assign short-read (MEGAHIT) and long-read (OPERA-MS and DBG2OLC) assemblies into GBs. Contamination of GBs in the DBG2OLC assembly was higher than in the other two assemblies. Accordingly, we used DBG2OLC to re-assemble the short-read and long-read mapping to GB.

**FIGURE 4:**
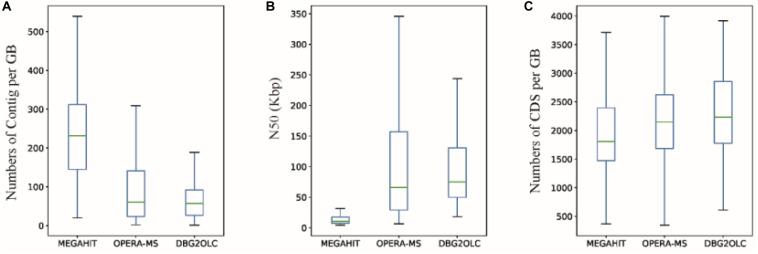
Characteristics of genome bins (GBs) reconstructed from different assemblies. **(A)** Number of contigs, **(B)** N50, and **(C)** number of coding sequences (CDS) per GB based on MEGAHIT short-read assembly and hybrid assemblies using OPERA-MS or DBG2OLC.

Finally, we obtained 132, 172, and 84 GBs using MEGAHIT, OPERA-MS and DBG2OLC, respectively, that were >60% complete and had <20% contamination. 78.57% of GBs (66) from DBG2OLC (64.53% for OPERA-MS) with ≤100 contigs were obtained, compared to 13.43% for MEGAHIT (Supplementary Tables S3–S5), which is an improvement over recently published approaches (Parks et al., 2017). The median number of contigs per GB for either OPERA-MS or DBG2OLC was only approximately 60 vs. 230 for MEGAHIT (Figure 4A), with 1 GB (MD001) with a single contig and 100% completeness. Compared to the GBs based on short-read assembly, the median N50 dramatically increased by ∼700% (Figure 4B), indicating that the incorporation of metagenomic data from the PacBio platform can greatly enhance the GB completeness. Incorporating PacBio long reads also led to more genes per GB compared to HiSeq-only assembly (Figure 4C and Supplementary Tables S3–S5). Median genome completeness was improved to ∼86.5% for OPERA-MS and DBG2OLC vs. 84.5% for MEGAHIT, with 2 GBs with 100% completeness and <1% contamination after incorporating long reads (for both OPERA-MS and DBG2OLC) vs. 0 GBs for MEGAHIT (Supplementary Tables S3–S5).

The 84 GBs from DBG2OLC were then used for further taxonomic classification, with 74 GBs belonging to bacteria and the remaining 10 belonging to archaea (Supplementary Table S3). Fourteen GBs could not be identified at the phylum level, indicating that many organisms in anaerobic digestion systems are still unknown. It is worth noting that we reconstructed a complete genome (MD001) with a single contig containing 1,520,274 bp and a GC content of 41.96% (Supplementary Figure S2A). AMPHORA2 was used for taxonomic classification, revealing that the complete genome belongs to the *Methanobacteriaceae* family. Metabolic reconstruction only indicated that MD001 is involved in the hydrogenotrophic pathway (Supplementary Figure S2B). Thus, PacBio long-read sequencing provides the possibility of studying unknown microorganisms.

## Discussion

In this study, we evaluated a short-read metagenomic assembler (MEGAHIT), two hybrid metagenomic assemblers (DBG2OLC and OPERA-MS) and depth-based hybrid assembly based on metagenomic data obtained using two sequencing approaches (Illumina HiSeq and PacBio). Hybrid assemblies using PacBio long reads and HiSeq contigs produced improvements in assembly statistics, including an increase in the average contig length, contig N50 size, and number of large contigs, which were also reported by other studies (Campanaro et al., 2016; Treu et al., 2016; Grohmann et al., 2018). However, hybrid methods have an inherent disadvantage regarding metagenomic assembly, e.g., need deep-sequencing data to ensure accuracy. Assembly of PacBio long reads only was attempted using Canu, but without success, presumably due to high-complexity metagenome samples. We did not attempt other assembly algorithms for assembly of PacBio long reads only or hybrid assembly of short-reads and long-reads.

We obtained a reference gene catalog (RGC) containing ∼2.66 million NR ORFs with an average length of 581 bp based on the three gene catalogs (Table 1). We observed PacBio long-read sequencing leading to more complete or unknown gene profiling of metagenomic samples. Strangely, the average gene length was shorter for DBG2OLC than for OPERA-MS and MEGAHIT, which may have been due to the samples containing large numbers of archaea with high GC content combined with DBG2OLC being more insensitive to high GC sequences (to our knowledge). More research is needed to optimize the hybrid assembly algorithm. Overall, further improvements of the sequencing technologies, the application of long-range information technologies combined with the development of new algorithms could greatly simplify the currently extensive assembly and polishing workflow.

Longer reads should eventually be able to overcome the need for binning approaches in more complex microbial communities. We also confirmed the feasibility of *de novo* assembling finished GBs incorporating long reads. The reconstruction of finished GBs was contributed to the comprehensive assessment of the overall functional potential of these microbial communities. The limitations to resolving finished GBs are defined by sequencing error rates and coverage, as the error rates in long reads precludes the resolution of similar genomes, and hampers assembly utility for downstream applications.

## Conclusion

Using different short- and long-read assemblers for metagenomic analysis of samples from an anaerobic digestion systems indicates that PacBio long reads improve metagenomic assemblies, gene catalog statistics, and genome binning and help to advance the functional understanding of microbiomes from anaerobic digestion systems. In particular, we constructed an anaerobic digestion microbiome gene catalog containing 2,662,347 NR ORFs, which has potential value for research on the functions of anaerobic digestion systems. We concluded that PacBio long reads produced longer contigs, more complete genes, and better genome binning, thereby offering more information about metagenomic samples.

## Data Availability Statement

Sequence data were deposited at the Sequence Read Archive (SRA, NCBI) under Bioproject PRJNA594186.

## Author Contributions

All authors listed have made a substantial, direct and intellectual contribution to the work, and approved it for publication.

## Conflict of Interest

FL and HX were employed by the company PUROTON Life Technology Group Co., Ltd. TJ was employed by the company BGI-Shenzhen. YS was employed by the company Tianjin Biochip Co., Ltd. The remaining authors declare that the research was conducted in the absence of any commercial or financial relationships that could be construed as a potential conflict of interest.

## Footnotes

1 https://www.pacb.com/support/software-downloads/

2 https://sourceforge.net/projects/amos/
