# Supplementary material for: PacBio Long Reads Improve Metagenomic Assemblies, Gene Catalogs, and Genome Binning
Source: Front Genet. 2020 Sep 8;11:516269. doi: 10.3389/fgene.2020.516269 (PMC7506068; doi:10.3389/fgene.2020.516269)
Supplement: Supplementary file 2 [file Data_Sheet_2.docx]

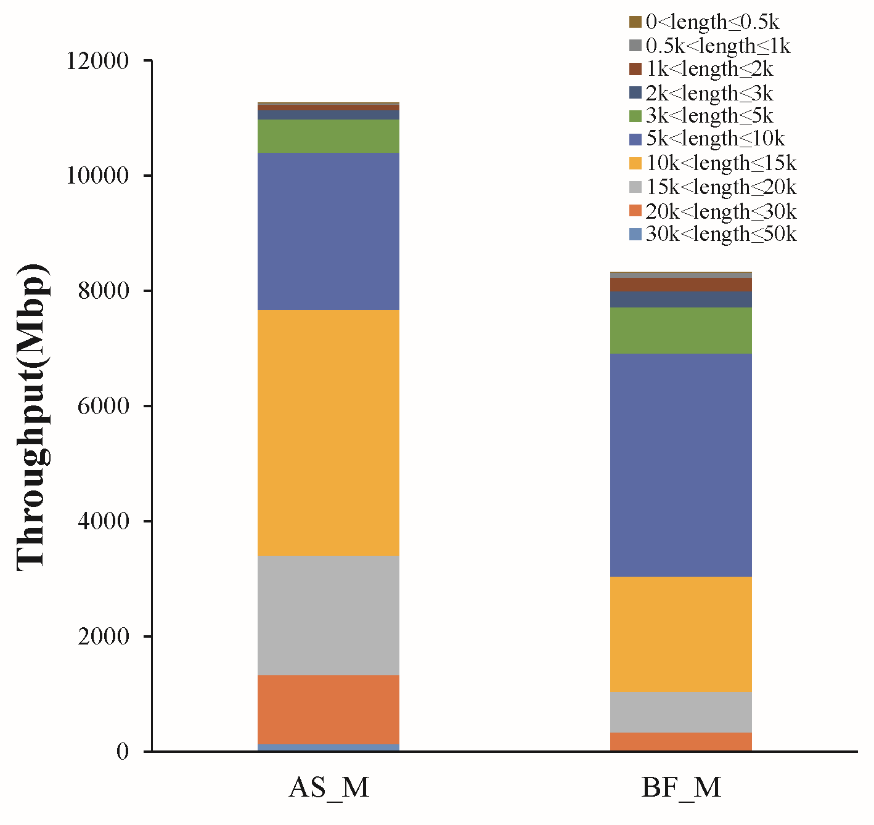


**Supplementary Figure 1:** Throughput function of PacBio long read length for the two samples. Reads >5 kbp provided >80% of the data in both samples. AS_M: Suspended Activated Sludge; BF_M; Biofilm on Carbon Fiber Filler.


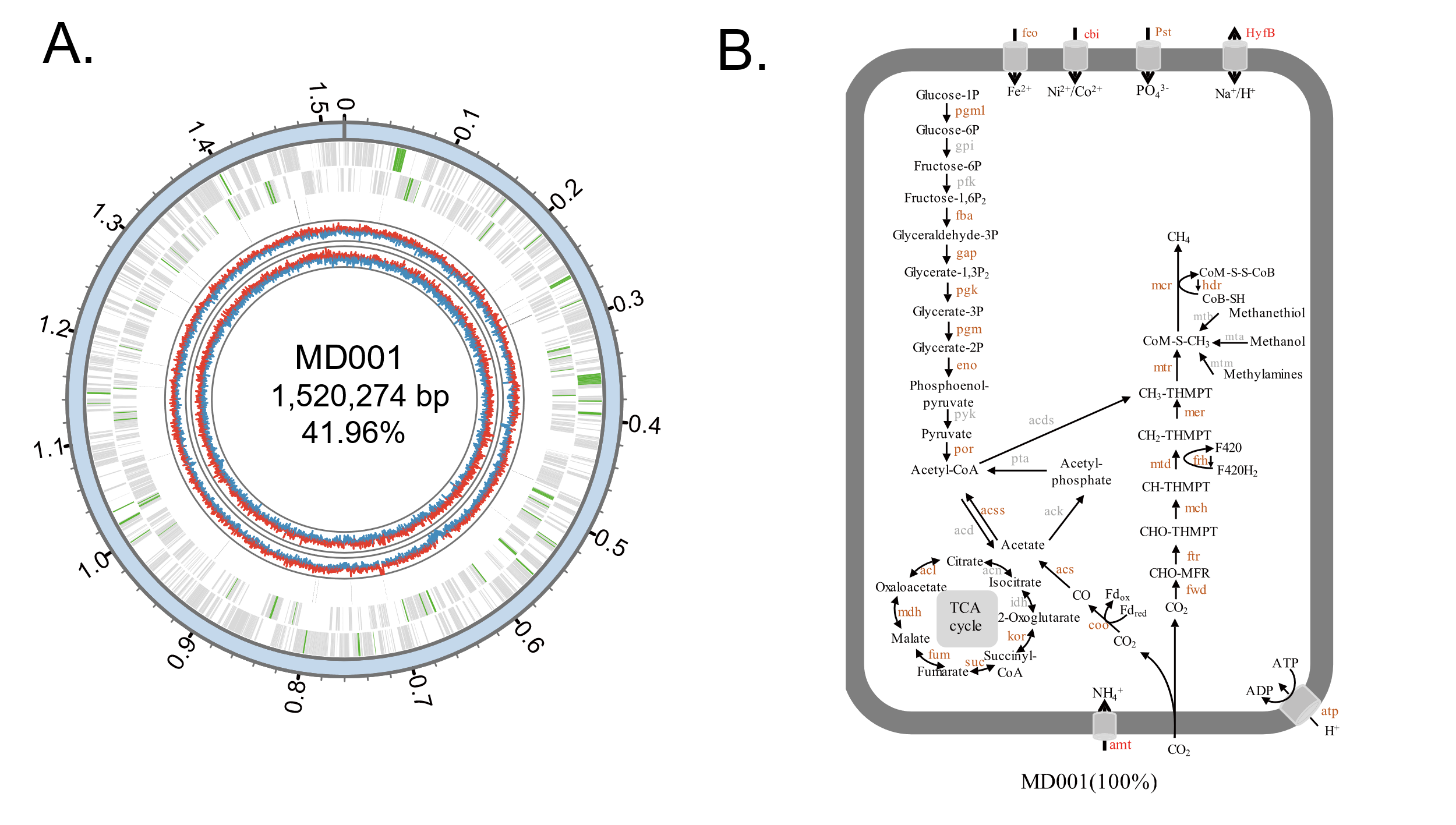


**Supplementary Figure 2:** Overview of draft genome and pathway reconstruction in MD001. **(A)** Binning draft genomes based on metagenomic sequences. Band 1—numbers of contigs, digits: bases (Mb); band 2—sense strand; band 3—antisense strand; band 4—tRNA and rRNA; band 5—GC content; band 6—GC skew. Gray indicates genes; green indicates genes in the methane pathway. **(B)** Pathway overview. Red indicates genes identified in the genome; grey indicates genes not identified in the genome.
